# Ultrathin Ge-YF_3_ antireflective coating with 0.5 % reflectivity on high-index substrate for long-wavelength infrared cameras

**DOI:** 10.1515/nanoph-2024-0360

**Published:** 2024-08-27

**Authors:** Jae-Seon Yu, Serang Jung, Jin-Woo Cho, Geon-Tae Park, Mikhail Kats, Sun-Kyung Kim, Eungkyu Lee

**Affiliations:** Department of Applied Physics, Kyung Hee University, Yongin-Si, Gyonggi-Do, 17104, Republic of Korea; Department of Electronic Engineering, Kyung Hee University, Yongin-Si, Gyonggi-Do, 17104, Republic of Korea; Department of Electrical and Computer Engineering, University of Wisconsin-Madison, Madison, WI, 53706, USA

**Keywords:** long-wavelength infrared cameras, discrete optimization, multilayer coating, binary optimization, antireflective coating

## Abstract

Achieving long-wavelength infrared (LWIR) cameras with high sensitivity and shorter exposure times faces challenges due to series reflections from high-refractive index lenses within compact optical systems. However, designing effective antireflective coatings to maximize light throughput in these systems is complicated by the limited range of transparent materials available for the LWIR. This scarcity narrows the degrees of freedom in design, complicating the optimization process for a system that aims to minimize the number of physical layers and address the inherent large refractive mismatch from high-index lenses. In this study, we use discrete-to-continuous optimization to design a subwavelength-thick antireflective multilayer coating on high-refractive index Si substrate for LWIR cameras, where the coating consists of few (e.g., five) alternating stacks of high- and low-refractive-index thin films (e.g., Ge-YF_3_, Ge-ZnS, or ZnS-YF_3_). Discrete optimization efficiently reveals the configuration of physical layers through binary optimization supported by a machine learning model. Continuous optimization identifies the optimal thickness of each coating layer using the conventional gradient method. As a result, considering the responsivity of a LWIR camera, the discrete-to-continuous strategy finds the optimal design of a 2.3-μm-thick antireflective coating on Si substrate consisting of five physical layers based on the Ge-YF_3_ high-low index pair, showing an average reflectance of 0.54 % within the wavelength range of 8–13 μm. Moreover, conventional thin-film deposition (e.g., electron-beam evaporator) techniques successfully realize the designed structure, and Fourier-transform infrared spectroscopy (FTIR) and thermography confirm the high performance of the antireflective function.

## 1 Introduction

Long-wavelength infrared (LWIR) cameras are increasingly in demand for a variety of applications, ranging from biomolecular spectroscopy for precisely analyzing protein folding and dynamics [1], [2], to drone-based surveillance for remote explosive detection [3], [4], and thermal objective detection for automotive and aviation safety [5], [6], [7]. Recently, these applications have necessitated not only more compact sizes but also higher resolution imaging capabilities in LWIR cameras, typically defined by their ability to distinguish fine details in thermal images even under variable environmental conditions [8], [9], [10]. This need for high-resolution is coupled with the requirement to maintain high sensitivity (i.e., a low noise equivalent temperature difference) at shorter exposure times, ensuring rapid and accurate detection and analysis. To meet these requirements, an optical system (e.g., a compound lens or multi-lens system), usually integrated in front of the camera, should have near-zero surface reflectance [11], [12]. Additionally, while the refractive index of each optical element should be higher to enhance the light-bending capability and thereby potentially reduce the overall size of the optical system, this configuration leads to an inherent increase in surface reflectance [13], [14]. Therefore, it is necessary for each optical element, such as a high-refractive-index lens, to be covered with a high-performance antireflective multilayer coating to optimize performance, ensuring maximum light throughput and minimizing losses due to reflection.

However, the limited availability of suitable materials for the LWIR complicates the practical design of antireflective coatings [13], [14], [15]. For example, while materials such as Ge, YF_3_, ZnSe, Si, and ZnS are available, practical considerations like minimizing the number of physical layers, reducing the variety of materials used, and decreasing the total thickness of the coating are essential [16], [17]. These factors are crucial not only for reducing thermal stress but also have to minimize the large refractive index mismatches from high refractive index lenses, which collectively hinder the systematic design of LWIR antireflective coatings [18], [19]. Additionally, the consideration of the spectral responsivity of photodetectors in the camera introduces further complexity. To address the issue, various optimization strategies have been proposed [20], [21], [22], [23], [24], [25]. For example, Moghadam et al. [20] used a gradient-based optimization package (Essential Macleod Program) to design a 2.24-μm-thick antireflective ZnS/Ge/ZnS/Ge coating, exhibiting the average reflectance of 2.86 % for the wavelength of 8–12 μm. Tikhonravov et al. [21] used a “needle optimization combined with gradual evolution technique” to optimize the antireflective multilayer coatings with ZnSe and YF_3_ materials. They found a 3.5-μm-thick coating with an average reflectance of 0.92 %, consisting of nine layers. Matsuoka et al. [22] used a modified conventional optical coating theory based on the effective refractive index change. They discovered a 2.5-μm-thick antireflective multilayer coating comprising ZnS/Ge/ZnS/YF_3_ layers on an InP substrate, showing an average reflectance of 0.62 % for wavelengths between 7 μm and 12 μm. While these approaches show promising performance, the optimized coatings exhibited a somewhat high average reflectance, used three or four different materials, or included several physical layers. In addition, they may not represent the optimal structure considering the responsivity of the photodetector and blackbody radiation [26], [27], [28] in the LWIR cameras.

Recently, numerous approaches leveraging machine learning techniques (e.g., deep neural networks, reinforcement learning, and inverse design) to optimize nanophotonic structures have been presented [29], [30], [31], [32], [33], [34], [35], [36]. Specifically, the design task of photonic structures has been successfully transformed into discrete (or binary) optimization problems [29], [31], [32], [33], [34], [35], [36], identifying optimal designs in applications such as monochromatic antireflective coatings for deep-ultraviolet photolithography [33], ultrathin optical diodes [34], radiative coolers [35], and digital metasurfaces for 5G telecommunications [36]. This process involves discretizing the photonic structure into a number, *N*, of domains, encoding each domain as a binary digit based on the material status, and then mapping the structure into a binary vector of length *N*. While conventional binary optimization techniques such as genetic algorithms [37], discrete particle swarm optimization [38], [39], and conformational space annealing [40], [41] have been used to optimize the figure-of-merit (*FoM*), the use of machine learning approaches, like factorization machines (FMs) [42], has proven particularly efficient in reducing computational costs. For example, Kitai et al. [43] and Kim et al. [33] demonstrated that using a training dataset encompassing only about 10^−9^ percent of the total candidates (2^*N*^), FMs-aided binary optimization could successfully identify locally optimal structures with promising *FoMs* for applications, such as radiative coolers [35] or optical diodes [34]. Kim et al. [33] suggested that these identified locally optimal structures serve as effective initial configurations for gradient-based methods to further refine continuous structural parameters, like the thickness of each physical layer in multilayer coatings. Additionally, these studies have revealed that the binary optimization can identified a substantial number of locally optimal structures with similar yet sufficiently good *FoMs*, which are close to the global minimum (or maximum). This offers flexibility in photonics design, allowing for the selection of relatively simple structures for fabrication. This indicates that a vast design space is available to systematically explore optimal antireflective coating designs, while addressing the practical factors with restricted material choices.

In this study, we employed a discrete-to-continuous optimization approach to design an antireflective multilayer coating based on two materials on a high-refractive-index Si substrate, tailored for LWIR cameras. The coating consists of a few alternating layers, each made from materials with high-and-low-refractive indices. We investigated three distinct high-low index pairs (Ge-YF_3_, Ge-ZnS, and ZnS-YF_3_) to determine the optimal structure of the coating. Within each high-low index pair, discrete optimization (DO) employing active learning via factorization machines efficiently identified the optimal configurations (e.g., physical number) of multilayer within the structural constraints (e.g., the minimal thickness of a film, and total thickness of the coating). Continuous optimization (CO) then quickly refined the thickness of each layer, maximizing the antireflective performance tailored to the LWIR camera. As a representative case, we discovered a 2.3-μm-thick antireflective coating comprising five physical layers based on Ge and YF_3_. In the best-case scenario, this optimal coating demonstrated an average reflectance of 0.54 %, with minimized reflectance occurring in wavelength regions where the responsivity of the LWIR camera is relatively high. We experimentally validated the performance of the designed antireflective coating on a Si substrate using Fourier-transform infrared (FTIR) spectroscopy and thermography.

## 2 Results and discussion

**Figure 1:**
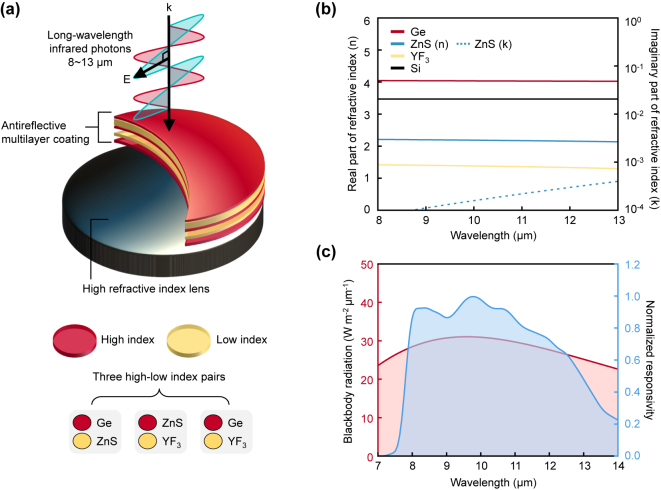
Design scheme of an antireflective multilayer coating for long-wavelength infrared (LWIR) camera applications. (a) Schematic of an antireflective multilayer coating on a silicon substrate. The high (or low) index layer is illustrated as a red (or yellow) planar layer. The coating consists of one of the three high-low index pairs: Ge-YF_3_, Ge-ZnS, and ZnS-YF_3_. (b) The real part and imaginary part of the refractive indices of Ge, ZnS, YF_3_, and Si as a function of wavelength. (c) The blackbody radiation spectrum (shaded with light pink) at 300 K and the normalized responsivity of the LWIR camera (shaded with light blue) as a function of wavelength.

The antireflective multilayer coating designed for LWIR cameras comprises two materials with alternating stacks of high-low-index thin-film layers (Figure 1a). We consider three different cases of high-low-index pairs: Ge-YF_3_, Ge-ZnS, and ZnS-YF_3_, as these materials show good transparency within 8 μm–13 μm, with negligible imaginary parts of refractive indices (see Figure 1b) [13], [14]. They can be also prepared using a conventional vacuum deposition process (e.g., electron-beam evaporator). For a given high-low index pair, we aim to identify the optimal configuration of the number of thin-film layers and their associated thickness with considering the practical factors. In optimization, a *FoM* can be defined to quantify the performance of the antireflective function tailored to the LWIR cameras, given by

(1) $$FoM=1-\frac{\overset{\lambda =13\mu \text{m}}{\underset{\lambda =8\mu \text{m}}{\int }}\bar{R}\left(\lambda \right)B\left(\lambda \right)T\left(\lambda \right)\text{d}\lambda }{\overset{\lambda =13\mu \text{m}}{\underset{\lambda =8\mu \text{m}}{\int }}\bar{R}\left(\lambda \right)B\left(\lambda \right)\text{d}\lambda },$$

where $T\left(\lambda \right)$ represents the transmittance of the antireflective multilayer coating in the 8 μm–13 μm wavelength region, $B\left(\lambda \right)$ denotes the blackbody radiation spectrum at 300 K, and $\bar{R}\left(\lambda \right)$ is the normalized responsivity of the LWIR camera (see Figure 1c). In Equation (1), a lower *FoM* indicates better antireflective performance; for example, *FoM* = 0 represents the ideal antireflective multilayer coating for the LWIR cameras. Thus, in the best-case scenario, the optimal configuration of the antireflective multilayers exhibits the lowest *FoM* within the design space.

**Figure 2:**
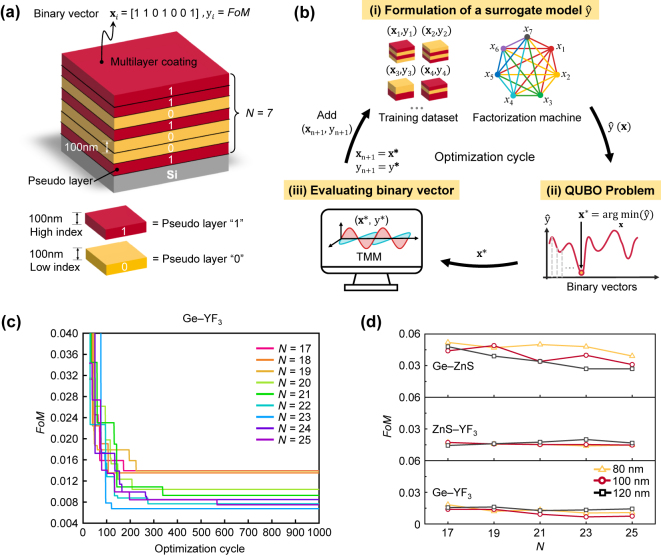
Discrete optimization (DO) scheme for the antireflective multilayer coating. (a) Schematic illustration of encoding a multilayer structure into a binary vector. For example, this case shows *N* = 7. The red (or yellow) planar layer with a binary digit “1” (or “0”) depicts a 100-nm-thick high (or low) index layer, also indicated as a pseudo-layer with “1” (or “0”). **x**_*i*_ and *y*_*i*_, respectively, depict a binary vector and an associated *FoM*. (b) The optimization cycle consists of three steps. Each step is explained in the main text. In step (i), *x*_*m*_ is the *m*th element of the binary vector **x**_*i*_. (c) The minimum *FoM* discovered as a function of optimization cycles at various *N* for the Ge-YF_3_ case. (d) The identified *FoM* as a function of *N* at the pseudo-layer thicknesses of 80, 100, and 120 nm for the Ge-ZnS pair, ZnS-YF_3_ pair, and Ge-YF_3_ pair.

A discrete-to-continuous optimization method was used to efficiently identify the optimal structure. This method was successfully applied to the design of a monochromatic antireflective coating for a deep ultraviolet wavelength of 193 nm [33]. In DO, the antireflective multilayer coating is discretized into 100-nm-thick layers, with each layer (the so-called pseudo layer) represented by a binary value (0 or 1), indicating the material (high or low index material). This representation yields a binary vector length of *N* (refer to the “*i*” step in Figure 2a), and wave optics simulations, such as the transfer matrix method [44], evaluates the *FoM* for each binary vector. With ‘*N*’ pseudo-layers, the optimal binary vector in the 2^*N*^ possible binary vectors had a *FoM* near the (local) minimum value of the *FoM* space. In other words, the antireflective multilayer coating corresponding to the optimal binary vector represents a quasi-optimal configuration. The CO uses this quasi-optimal configuration as an initial point to refine the thickness of the physical layers and reach the local minimum value of the *FoM*. In short, DO aims to determine the optimal configuration of physical films, while CO identifies the optimal thickness of each physical layer (see the Supporting Information for details of the DO combined with CO method).

However, discovering the optimal binary vector using wave-optics simulations can be computationally expensive, especially as the length of the binary vector increases. To address this challenge, the DO utilizes an active learning scheme. In active learning, an initial training dataset comprising 25 binary vectors and their associated *FoMs* is prepared. With the initial training dataset, the active learning scheme is performed through iterative cycles, with each cycle consists of the following three steps (see Figure 2b): (i) A factorization machine (FM) [42] formulates a surrogate model using the training dataset. A binary vector is defined as $x_{i}\left(x_{i}\in {\left\{0,1\right\}}^{N},i=1,\ldots ,n\right)$ , and *FoMs* is depicted by *y*_*i*_ ( $y_{i}\in R$ ). Then, the surrogate model $\hat{y}$ can be expressed as:

(2) $$\hat{y}\left(x\right):=w_{0}+\overset{N}{\underset{i=1}{\sum }}w_{i}x_{i}+\frac{1}{2}\overset{k}{\underset{f=1}{\sum }}\left({\left(\overset{N}{\underset{i=1}{\sum }}v_{i,f}x_{i}\right)}^{2}-\overset{N}{\underset{i=1}{\sum }}v_{i,f}^{2}x_{i}^{2}\right),$$

where *w*_0_ is the global bias, *w*_*i*_ is the linear strength, and *v*_*i*,*f*_ is the latent vector of size *N* × *k*, which defines the quadratic interaction between *i*th (*x*_*i*_) and *j*th (*x*_*j*_) elements of **x** (*k* = 8 is fixed in this study). (ii) An optimal binary vector that minimizes the surrogate model is identified using quadratic unconstrained binary optimization (QUBO) [45]. There are several strategies for solving QUBO, such as Ising machines [46], simulated annealing [47], quantum annealing [48], [49], [50], particle swarm methods [38], [39], Gurobi [51], and exhaustive enumeration. In this study, we employ the exhaustive enumeration method for *N* ≤ 22 and switch to quantum annealing for *N* ≥ 23 to identify the optimal binary vector for the surrogate model. It is important to note that while exhaustive enumeration accurately identifies the global solution of the QUBO problem, its computational time is severely constrained by memory capacity. In our computational resources, the performance of exhaustive enumeration significantly degrades when *N* ≥ 23, leading us to adopt quantum annealing via the D-wave leap platform for these cases [49], [50]. (iii) The identified optimal binary vector is evaluated by wave optics simulations and added to the training dataset. If the identified optimal binary vector already exists in the training dataset, a new binary vector is randomly generated and added. The iterative cycles of the DO process were suspended when the number of optimization cycles reached 1,000.

In the DO process, the thickness per bit and length of the binary vectors should consider the practical factors. In our case, the minimum thickness of the thin film layer should be greater than ∼70 nm to minimize the variation of refractive index depending on the thickness (see Figure S1 in Supporting Information). Additionally, the references [20], [21], [22] show that it is advisable to limit the thickness of the coating to less than ∼3 μm for mechanical stability. Thus, we set the thickness per bit to 80 nm, 100 nm, and 120 nm, with the length of the binary vectors ranging from 17 to 25 in the DO process. These various thickness-per-bit values can efficiently search the quasi-optimal structure in the *FoM* space. Under these structural constraints, we observed that the DO could discover locally optimized binary vectors within the optimization cycle of 300–500, corresponding to 0.0015–0.14 % of possible binary states (see Figures 2c and S2 in Supporting Information). For all three high-low-index pairs, the Ge-YF_3_ case shows excellent antireflective performance (see Figure 2d). In addition, for each high-low index pair, the distribution of quasi-optimal *FoMs* discovered through the DO process were found in a similar range across different thickness-per-bit values of 80, 100, and 120 nm. In the best case, the optimal coating with Ge-YF_3_ films had an *FoM* of 0.0067 at *N* = 23 with a thickness per bit value of 100 nm. This value is 3.3 % (or 0.9 %) lower than the best case of the Ge-ZnS pair (or ZnS-YF_3_ pair). These results let us select the Ge-YF_3_ films with the thickness-per-bit value of 100 nm for the CO optimization. It is noted that the quality of initial training datasets can influence the early stages of the DO optimization cycles, resulting in variations in the identified binary vectors. However, at higher optimization cycles, the DO consistently identifies the same optimal binary vector, indicating that the effect of initial conditions on the performance of the DO is minimal (see Figure S3 in Supporting Information).

**Figure 3:**
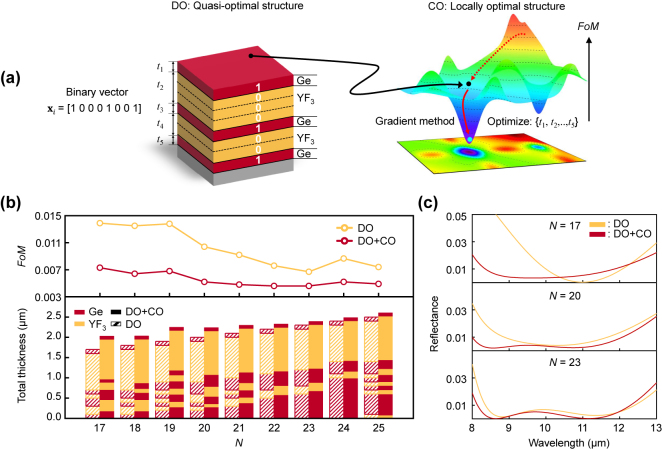
Continuous variable optimization (CO) after the DO process. (a) (Left) Schematic illustration defining the physical layers and the thickness from the quasi-optimal structure identified by the DO process. The dotted lines indicate the boundaries of the pseudo-layers. *t*_*i*_ depicts the thickness of each physical layer. (Right) Schematic of the *FoM* parametric space. The black point indicates the position of the quasi-optimal structure. The red solid (or dash) line indicates the history of optimization by the CO (or DO) process. (b) (Top) The identified *FoM* as a function of *N* and (Bottom) the corresponding structure of the Ge-YF_3_-based antireflective coating with or without the CO process. (c) The calculated reflectance as a function of wavelength optimized at various *N* values with or without the CO process.

The DO process reveals the optimal configuration of physical thin-film layers at each value of *N*. Subsequently, the multilayer coating discovered by DO can be described by an *m*-number of physical layers, each with an initial thickness *t*_*i*_ (see Figure 3a). An interior-point algorithm was employed to refine the thickness of each layer, subject to the constraints *t*_*i*_ − 80 nm < *t*_*i*_ < *t*_*i*_ + 80 nm (if *t*_*i*_ > 160 nm) or 80 nm < *t*_*i*_ < *t*_*i*_ + 80 nm (if *t*_*i*_ < 160 nm), to limit the maximum thickness of the coating. From Figure 3b, it is evident that CO further improves the *FoM* of the quasi-optimal structure in all cases. Interestingly, the lower *N* cases demonstrated a significant improvement in *FoM* with CO. For example, the quasi-optimal structures with *N* values of 17–19 had *FoMs* of 0.014, which are reduced to 0.006–0.008 with the CO. The reduction gradually decreased as increasing *N*, and at *N* = 23, the *FoM* of 0.0067 was reduced to 0.0046. Also, the reflectance spectra with or without the CO process clearly show these improvement characteristics (see Figure 3c). At *N* = 17, there is a noticeable reduction in the reflectance spectrum with the CO process across 8–13 μm wavelengths. However, at higher *N*, the reflectance spectrum without the CO process (i.e., the DO-optimized structure) is very similar to that with the CO process. These results imply that a quasi-optimal structure with a higher *N* is closer to the local optima in the *FoM* space. It is noted that the performance of CO strongly depends on the quality of the initial point. For example, if the initial points are randomly given (which may be far from the optimal point and of poor quality), the structures identified by the CO have higher *FoMs* than those identified by the DO combined with the CO approach (see Figure S4 in the Supporting Information). The CO-optimized structures exhibited similar *FoMs* values, but their structural configurations varied. This shows that the *FoM* space contains many local optimal points that are effectively identified through the DO combined with the CO strategy, well corresponds with the previous studies [33]. Notably, among the various optimal structures in Figure 3b, we selected the CO-optimized structure at *N* = 23 for better fabrication and further study, as it exhibits the lowest *FoM* of 0.0046 with fewer physical layers (80-nm Ge/1080-nm YF_3_/348-nm Ge/205-nm YF_3_/680-nm Ge) and a total thickness of 2.39 μm.

**Figure 4:**
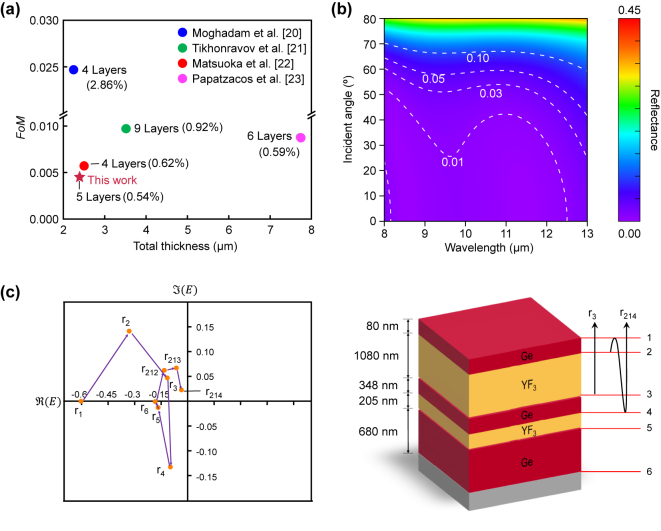
Characteristics of an optimized Ge-YF_3_ antireflective multilayer coating. (a) Comparison of the *FoM* of the coating in this study with references [20], [21], [22], [23]. The value in a parenthesis is the averaged reflectance. (b) The reflectance as a function of wavelength at various incident angles of the coating. (c) (Left) The phasor analysis of the coating at the wavelength of 8.6 μm at the incident angle of 0°. (Right) Schematic illustration of the reflection coefficients of *r*_*i*_ and *r*_*ijk*_ in the phasor analysis, where *i*, *j*, or *k* is the index of optical interface. The red solid lines depict the optical interfaces in the coating, where each interface is labeled as 1–6 from the top to the bottom.

The selected CO-optimized structure at *N* = 23 exhibited the lowest *FoM* as well as the averaged reflectance compared to the reported antireflective multilayer coatings, highlighting the effectiveness of the DO-combined with CO optimization (see Figure 4a). We also investigate the reflectance of this structure as a function of wavelength (8–13 μm) and incident angles to study the fundamentals of the antireflective function (see Figure 4b). In the 8–13 μm wavelength region, the reflectance spectrum at normal incidence exhibits a ‘W-like’ shape (see Figure 3c), where the half-maximum of reflectance is ∼0.0051. The double valleys (i.e., the wavelengths at the minimum of reflectance) are at wavelengths of 8.64 μm and 11.16 μm, respectively. Additionally, the coating showed relatively low reflectance values in the wavelength region (8.5–11 μm) where the responsivity of the LWIR camera is high. Furthermore, this reflectance spectrum is nearly maintained up to an incident angle of 50°, which is in contrast to that of a conventional silicon substrate (see Figure S5 in Supporting Information). Such wide-angle antireflective performance can potentially benefit the design high numerical aperture lens [52], [53].

For further analysis, we examined the phasor diagram of the optimized structure at a valley of 8.6 μm (Figure 4c). Because the structure comprises seven optical layers, including the top air and bottom Si substrates, there are six optical interfaces. The first-order reflection coefficients (*r*_1_ to *r*_6_) for each interface were calculated, and their accumulative summation in the complex plane is shown in the phasor diagram of Figure 4c. The subscript of the reflection coefficient is ordered from the top to the bottom interface (e.g., *r*_1_ corresponds to the reflection coefficient at the air/85-nm-thick Ge layer interface). The addition of these reflection coefficients resulted in a net vector with an amplitude of 0.0338, which was further reduced to 0.0017 by adding the third-order reflection coefficients (e.g., *r*_212,_ *r*_213_, and *r*_214_, see Figure 4c for the definition of *r*_*ijk*_). The phasor analysis indicated that the primary factor contributing to the antireflective function was the first-order reflection coefficient at each interface, whereas further reduction was achieved when multiple reflections across three interfaces were accounted. In the DO process, these characteristics of first-order reflection coefficients may be captured and dumped on quadratic interactions in a surrogate model by the FM.

**Figure 5:**
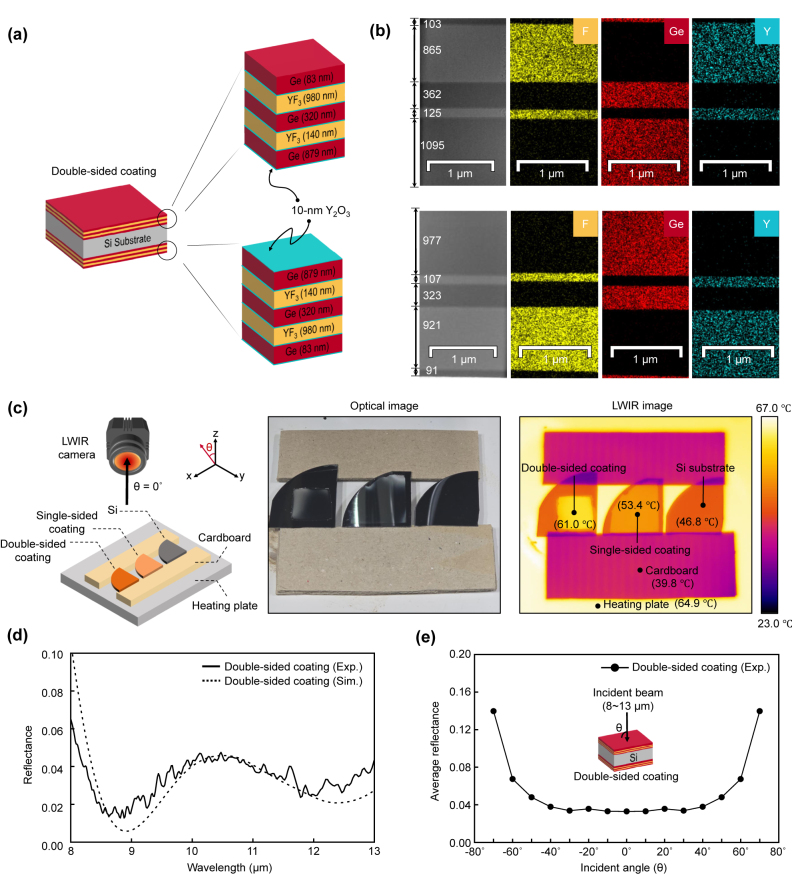
Experimental verification of the optimized Ge-YF_3_ antireflective multilayer coatings. (a) Schematic illustration of the double-side coating structure, where the antireflective multilayers are coated on the top and bottom of the Si substrate. It is noted that the sequence of physical layers on the bottom side is reversed. The thickness of each layer is obtained from the DO combined with CO process. (b) The cross-sectional TEM and EDX images of fabricated double-sided coating. The thickness of each layer is obtained from the TEM images. The yellow, red, and blue areas indicate F, Ge, and Y, respectively. (Top) Top side of double-sided coating, (Bottom) bottom side of double-sided coating. (c) Comparison of antireflective performance in fabricated samples using LWIR thermography. (left) Schematic of the experimental setup. (middle) The optical image of fabricated samples. (right) The LWIR image of fabricated samples. (d) The reflectances of the double-side coating as a function of wavelength. Exp. (or Sim.) depicts that the reflectance is from the FTIR (or TMM). The simulated reflectance was obtained with modified complex refractive index of Si substrate. (e) The average reflectance of the double-side coating as a function of incident angles.

We experimentally verified the performance of the optimized Ge-YF_3_ antireflective multilayer coating. For fabrication, a 10-nm-thick Y_2_O_3_ adhesive layer [22] was introduced to enhance the mechanical adhesion between the Ge and YF_3_ layers. The DO combined with CO was used again to optimize the Ge-YF_3_ antireflective multilayer coating with a 10-nm-thick Y_2_O_3_ adhesive layer. The optimized structure (see Figure 5a) had an average reflectance of 0.62 %, which was similar to that without the Y_2_O_3_ adhesive layer, meaning the adhesive layer barely influenced the optimized performance. To reduce unwanted reflections from the bottom of the Si substrate and the air interface, optimized multilayer coatings were fabricated on both sides (top and bottom) of the Si substrate using an electron-beam evaporator (i.e., double-sided coating). Cross-sectional transmission electron microscope (TEM) images of the fabricated double-sided coating revealed that the thicknesses of the layers in the fabricated coating are slightly different from those in the designed one. These variations are due to fluctuations in the deposition rate during the thick-film growth process and alterations in the temperature of the substrate, which potentially lead to variations in the vapor density of the deposited materials [54]. Nonetheless, each layer was uniformly deposited while maintaining distinct mechanical interfaces (see Figure 5b). Energy-dispersive X-ray (EDX) spectroscopy images further confirmed that the Ge, YF_3_, and Y_2_O_3_ adhesion layers were well-deposited.

We performed LWIR thermography to verify the antireflective performance of the fabricated samples (see Figure 5c). The double-sided coating, single-sided coating, and a bare silicon substrate were placed between the LWIR camera and a heating plate set to 64.9 °C, with a cardboard spacing layer to avoid heat conduction. The LWIR images show that the temperatures of the heating plate passing through the double-sided and single-sided coatings are 61.0 °C and 53.4 °C, respectively, while that through a bare silicon substrate is 46.8 °C. We further investigated the reflectance of the double-sided coating using FTIR spectroscopy (see Figure 5d). The double-sided coating exhibited an average reflectance of 3.31 % at normal incidence, which, although slightly higher than the theoretical value of 1.09 %, confirmed the high-performance antireflective function of the designed multilayer coating. One factor contributing to the discrepancy in the reflectance value is the extinction coefficient of the silicon substrate, which can arise from the manufacturing process (i.e., mechanical dopants). When the imaginary parts of the refractive index of Si substrate were adjusted (*k* ∼ 10^−4^), the measured reflectance spectrum is matched well with the theoretical values. It is anticipated that using silicon lenses with an extinction coefficient near zero would improve antireflective performance in practice. Additionally, we investigated the reflectance of the double-sided coating in the wavelength range of 8–13 μm at various incident angles, from normal to 70°, using FTIR spectroscopy (see Figure 5e). The measurements show that the average reflectance of the double-sided coating at normal incidence is maintained up to an incident angle of 50°, exhibiting good agreement with the theoretical studies in Figure 4b.

## 3 Conclusions

This study optimized the antireflective multilayer coating using the combined DO and CO strategy, with thickness and material constraints for the LWIR cameras. The Ge-YF_3_ pair was selected as the representative case due to its better performance than the Ge-ZnS and ZnS-YF_3_ pairs. The optimized antireflective coating based on the Ge-YF_3_ pair demonstrated an *FoM* of 0.0045 (with an average reflectance of 0.54 %) at the wavelength of 8–13 μm. This high-performance antireflective function was experimentally verified using double-sided on a silicon substrate via FTIR and thermography. A functional multilayer coating (e.g., band-pass filter) requiring more than two material candidates can effectively leverage the DO combined with the CO strategy with longer-bit binary bases (e.g., 00, 01, 10, and 11). The accuracy of the surrogate model in the DO process can be further enhanced by leveraging higher-order FMs [55]. In addition, the *FoM* can be adequately adjusted to suit specific photonic applications. For example, while this study shows that the Ge-YF_3_ pair exhibits promising antireflective performance, it can potentially experience mechanical delamination due to the large mismatch in thermal expansion coefficients when the temperature varies. Therefore, considerations of built-in mechanical stress as a function of temperature within a multilayer structure can be integrated into the *FoM* definition. Practical applications can consider this mechanical stress to overcome the delamination issue.

## 4 Methods

### 4.1 Optimization

In the DO process, an FM model within the “xLearn” package was used to formulate a surrogate function. The hyperparameters (i.e., *w*_0_, *w*_*i*_, and *v*_*i*,*f*_) of the FM model were learned using the training datasets by minimizing the loss function through a stochastic gradient method with a learning rate of 0.001 and an L2 regularization parameter of 0.001, with epochs set at 20,000, and early stopping criteria. For *v*, the length of the latent vector *k* was fixed at 8. In the training dataset, 80 % of the dataset was used for supervised learning, and 20 % of the dataset was used for cross-validation. Active learning was performed using a workstation (AMD Ryzen™ Threadripper™ PRO 5995WX, 64-Cores, 512 GB). Quantum annealing was performed using a D-wave leap. In the CO process, an interior point algorithm (MATLAB optimization toolbox “fmincon”) was used. The CO was performed on a workstation (Intel (R) Core (TM) i5-12400F, 32GB).

### 4.2 Fabrication

The multilayer coatings were prepared on a silicon substrate (thickness 500 ± 30 μm, Hi-Solar Co. Ltd.). For optical and material characterizations, a thin film was deposited on a quartz substrate (thickness 500 ± 30 μm, Hi-Solar Co., Ltd.). The substrate (silicon or quartz) was sequentially cleaned with acetone, isopropyl alcohol, and distilled water using ultrasound, followed by a soft bake at 150 °C for 10 min to remove moisture and enhance adhesion. Thin films of Ge (iTasco, 99.99 %), YF_3_ (iTasco, 99.99 %), or Y_2_O_3_ (iTasco, 99.99 %) were deposited on the substrate using a conventional e-beam evaporator at 10^−6^ Torr, with a deposition rate of 3 Å/s for the Ge film, 5 Å/s for the YF_3_ film, and 1 Å/s for the Y_2_O_3_ film. During the deposition process of thin films, the substrate temperature was maintained at 120 °C.

### 4.3 Characterization

The refractive indices of YF_3_, Ge, ZnS, and Y_2_O_3_ thin films were obtained with ellipsometry (IR-VASE, J. A. Woollam Co.) in the 5–24 μm wavelength regions. The thicknesses of the thin films were measured using a surface scan profiler (Alpha-step D-500, KLA-Tencor). Reflectance spectra of the double-side antireflective coatings were recorded using an FTIR spectrometer (INVENIO R, Bruker) equipped with a gold diffuser-coated integrating sphere (A562-G/Q, Thorlabs) and an HgCdTE (MCT) detector (1.6–14 μm). The FTIR spectrometer, equipped with a variable angle reflection accessory (Seagull, Harrick), was employed to obtain the angle-resolved reflectance spectrum (2.5–24 μm). To minimize measurement errors caused by reflections from the sample loader during the measurement process, a black soot sample (*ε*_avg_ = 0.91) was applied to the loader as an absorber. Cross-sectional TEM and EDX images of the samples were obtained using a high-resolution transmission electron microscope (JEM-2100F, JEOL), and the samples were prepared using a focused ion beam (Nova 600, Nanolab). The LWIR thermography images were captured with an infrared camera (FLIR A655SC), which operates within a spectral range of 7.5–13.5 μm. Calibration of each image was performed using a silicon wafer coated with carbon tape as the reference standard.

### 4.4 Phasor analysis

In a multilayer system, partially reflected waves can be modeled as phasors. Despite the infinite number of partially reflected waves, the reflectance can be effectively approximated by considering only first-order reflections, which account for single instances of reflection. The complex amplitudes for reflection and transmission of these phasors were determined using Fresnel’s coefficients, denoted as *r*_*lm*_ and *t*_*lm*_ for reflection and transmission coefficients between layers *l* and *m*. Phase changes were calculated based on optical path differences, which depend on the transmitted angle *q*, layer thickness *d*, the refractive index *n* of medium, and the wave vector *k*_0_ of light source. In mathematical expression, optical path difference *L* when the light travels from layer *l* to *m* is given by Λ = 2*nk*_0_ *d* cos*θ*. Transmitted angle at each interface was individually determined using Snell’s law, which involves converting the transmitted angle at one interface to the incident angle at the subsequent interface. These calculations were performed analytically, employing a procedure that multiplies the amplitudes and sums the phase changes in a sequential manner, starting from air and proceeding to the substrate.

## Supplementary Material

Supplementary Material Details
