# Supplementary material for: Ultrathin Ge-YF3 antireflective coating with 0.5 % reflectivity on high-index substrate for long-wavelength infrared cameras
Source: Nanophotonics. 2024 Aug 27;13(21):4067–78. doi: 10.1515/nanoph-2024-0360 (PMC11501071; doi:10.1515/nanoph-2024-0360)
Supplement: Supplementary file 1 — Supplementary Material Details [file j_nanoph-2024-0360_suppl_001.pdf]

Supporting Information

Ultrathin Ge-YF_3 Antireflective Coating with 0.5% Reflectivity on High-Index Substrate for Long-wavelength Infrared Cameras

Jae-Seon Yu[1+], Serang Jung[2+], Jin-Woo Cho[3], Geon-Tae Park[1], Mikhail Kats[3], Sun-Kyung Kim[1*], Eungkyu Lee[2*]

[1] Department of Applied Physics, Kyung Hee University, Yongin-Si, Gyonggi-do, 17104, Republic of Korea

[2] Department of Electronic Engineering, Kyung Hee University, Yongin-Si, Gyonggi-do, 17104, Republic of Korea

[3] Department of Electrical and Computer Engineering, University of Wisconsin-Madison, Madison, WI, 53706, USA

[+] These authors contributed equally to this study.

[*]Corresponding authors: sunkim@khu.ac.kr, eleest@khu.ac.kr

DO combined with CO method

The DO starts with 25 randomly selected binary vector – *FoM* data pairs. Given the existence of an unknown function space that maps binary vectors to *FoM*s, calculating the *FoM* for all possible binary vectors using the transfer matrix method (TMM) is computationally prohibitive due to the exponential number of binary vectors (2^N). To address this, we construct a surrogate function using the available training data (10 to 1000 samples). The factorization machine (FM) is utilized to approximate the *FoM* for any binary vector, forming the surrogate function. FM captures the interactions between the elements of the binary vector, resulting in a representation that consists of an $N \times N$ matrix and a single bias term. This representation significantly reduces computational costs than evaluating TMM directly. Using the surrogate function, we optimize the binary vector to minimize the *FoM* (see Method section for details).

To find the optimal binary vector yielding the minimum output of the FM-based surrogate function, we use two approaches. The first method is exhaustive enumeration, which uses all 2^N possible binary vectors to obtain the minimum of output of the surrogate function formed by FM. The second approach is quantum annealing, which identifies the optimal binary vector with polynomial time complexity by using adiabatic annealing of entangled superconducting qubits. The FM model is expressed in the quadratic unconstrained binary optimization (QUBO) matrix form, translatable into the Hamiltonian of a quantum computer. Unlike classical bits, qubits in a quantum computer can exist in superposition states, enabling faster identification of the minimum point. For the efficient DO process, the quantum annealing is introduced for $N \geq 23$.

The binary vector identified by the surrogate function is then evaluated using TMM to determine its *FoM*. The newly identified binary vector and its *FoM* data are fed back into the training dataset, repeating the active learning cycle. This iterative process accumulates optimal structures with local or global minimum *FoM*s in the training data. Through sufficient iterations, a structure with a low *FoM* and suitable for experiment can be selected.

Following the discrete optimization (DO) process, the final structure has discrete thickness values, which may require further optimization. Therefore, an additional optimization process using the interior-point method is applied to continuously optimize the thickness. Interior-point method is the gradient-based algorithm designed for solving linear and nonlinear convex optimization problems [1,2]. A critical aspect of continuous optimization (CO) is the choice of the initial point; thus, the structure determined by DO is used as the initial point for CO (see Figure S4). In this study, the maximum allowed number of iterations is set to 1000, and the minimum allowed step size is set to 10^{-10}.

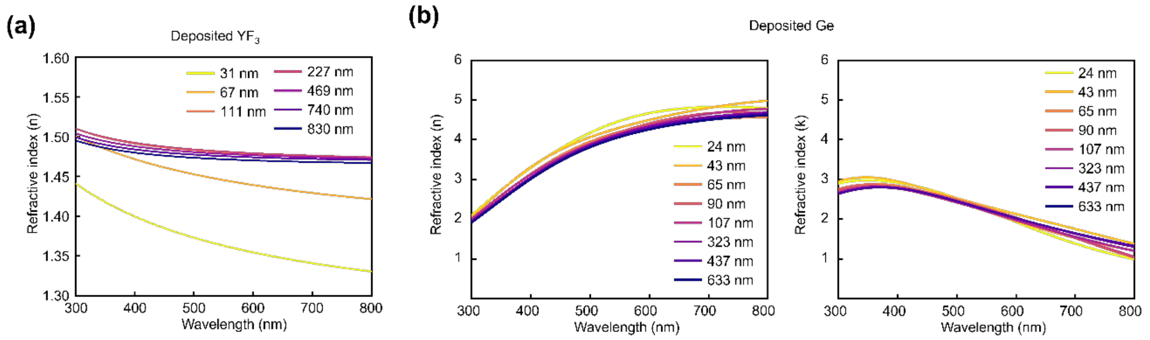

Figure S1| Refractive indices of Ge and YF_3 at various thickness deposited by e-beam evaporation. (a) Refractive indices of YF_3. (b)(left) The real part of refractive indices of Ge. (right) The imaginary part of refractive indices of Ge.

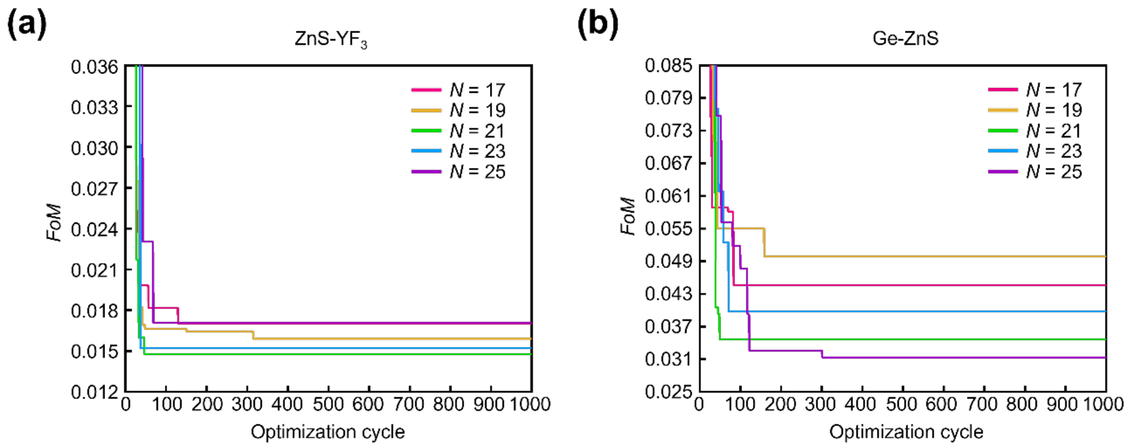

Figure S2| The minimum *FoM* discovered as a function of optimization cycles at various *N* for (a) ZnS-YF_3 pair, (b) Ge-ZnS pair. The pseudo-layer thickness is 100 nm.

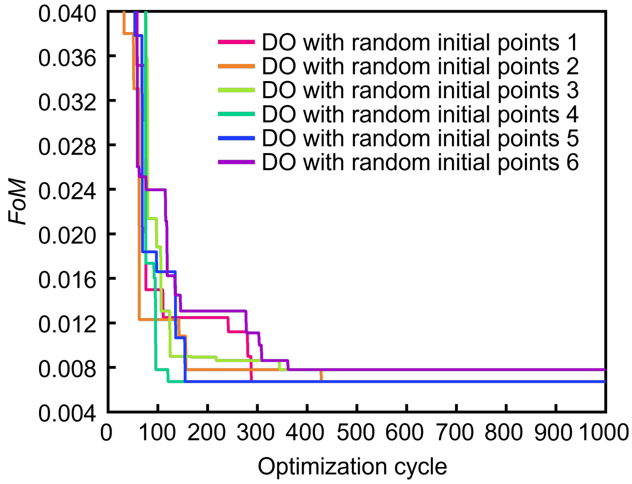

Figure S3| The minimum *FoM* discovered as a function of optimization cycles of the DO at various batches of the initial points, with Ge-YF_3 pairs at $N = 23$ with a thickness per bit value of 100 nm. Each initial points consists of 25 binary vectors and their associated *FoMs*. As a result of the DO processes, the lowest *FoM* of 0.0067 was found for all datasets except for initial point 6, which had an *FoM* of 0.0078.

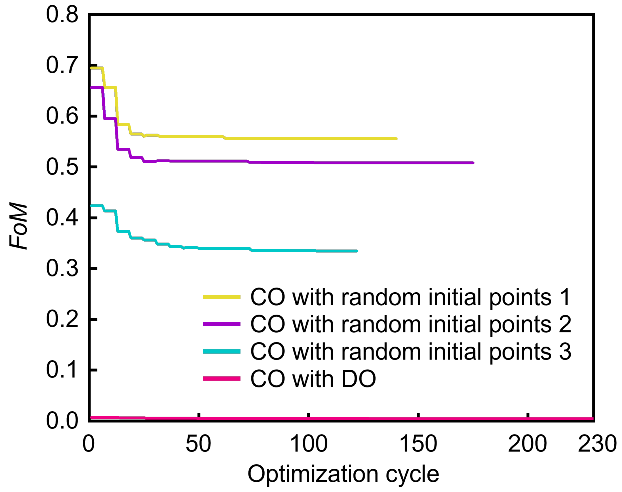

Figure S4| The *FoM* as a function of optimization cycles of the CO (i.e., interior points method) at various initial points for Ge-YF_3 pairs with 5 layers. The CO with DO case uses the DO-optimized structure at $N = 23$ bits as the initial point. The thickness of random initial points is composed of random integers between 100 nm and 1000 nm. The process of CO terminates when the early stopping criterion is satisfied.

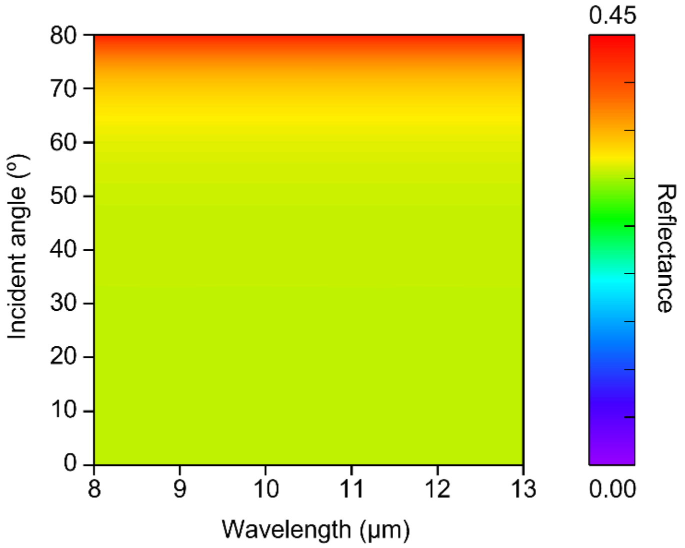

Figure S5| The reflectance as a function of wavelength at various incident angles of the Si substrate.

References

[1] F. A. Potra and S. J. Wright, "Interior-point methods," *J. Comput. Appl. Math.*, vol. 124, no. 1-2, pp. 281-302, 2000, https://doi.org/10.1016/S0377-0427(00)00433-7.

[2] N. Karmarkar, "A new polynomial-time algorithm for linear programming," Proc. 16th Annu. ACM Symp. Theory Comput., 1984, pp. 302-311, https://doi.org/10.1145/800057.808.
